# Transcriptomic Identification and Biochemical Characterization of HmpA, a Nitric Oxide Dioxygenase, Essential for Pathogenesis of *Vibrio vulnificus*

**DOI:** 10.3389/fmicb.2019.02208

**Published:** 2019-09-24

**Authors:** Dukyun Kim, Eun Jung Na, Suhyeon Kim, Jung Sung Kim, Young Hyun Jung, Jiafu Cao, Ho Jae Han, Iel Soo Bang, Jin-Wook Yoo, Nam-Chul Ha, Sang Ho Choi

**Affiliations:** ^1^National Research Laboratory of Molecular Microbiology and Toxicology, Seoul National University, Seoul, South Korea; ^2^Department of Agricultural Biotechnology, and Center for Food Safety and Toxicology, Seoul National University, Seoul, South Korea; ^3^Department of Microbiology and Immunology, Chosun University School of Dentistry, Gwangju, South Korea; ^4^Department of Veterinary Physiology, College of Veterinary Medicine, Research Institute for Veterinary Medicine, BK21 PLUS Creative Veterinary Research Center, Seoul National University, Seoul, South Korea; ^5^College of Pharmacy, Pusan National University, Busan, South Korea

**Keywords:** *Vibrio vulnificus*, microbiology, nitric oxide dioxygenase, flavohemoglobins, virulence factors, gene expression profiling

## Abstract

Nitric oxide (NO) and its derivatives are important effectors of host innate immunity, disrupting cellular function of infecting pathogens. Transcriptome analysis of *Vibrio vulnificus*, an opportunistic human pathogen, identified a set of genes induced upon exposure to NO. Among them, *VvhmpA* (*V. vulnificus hmpA*), encoding a multidomain NO dioxygenase, was the most greatly induced upon exposure to NO and was thus further characterized. Absorption spectra demonstrated that *Vv*HmpA is a heme protein in which the heme iron can exist in either reduced, NO-bound, or oxidized state. Biochemical studies revealed that *Vv*HmpA is a flavohemoglobin containing equimolar amounts of heme and FAD as cofactors. The *K*_M_ and *k*_cat_ values of *Vv*HmpA for NO at 37°C, the temperature encountered by *V. vulnificus* in the host, were greater than those at 30°C, indicating that *Vv*HmpA detoxifies high levels of NO effectively during infection. Compared with the wild type, the *VvhmpA* mutant exhibited a lower NO-decomposition activity and impaired growth in the presence of NO *in vitro*. Also, the cytotoxicity and survival of the *VvhmpA* mutant infecting the NO-producing murine macrophage cells were lower than those of the wild type. Furthermore, the mouse lethality of the *VvhmpA* mutant was reduced compared to that of the parental wild type. The combined results revealed that *Vv*HmpA is a potent virulence factor that is induced upon exposure to NO and important for the survival and pathogenesis of *V. vulnificus* during infection.

## Introduction

Nitric oxide (NO) and its derivatives, collectively called reactive nitrogen species (RNS), are among the most important components of the host innate immune system, the first line of defense against infecting pathogens (Fang, 2004). Under infectious conditions, NO is produced by inducible NO synthase (iNOS) which is expressed in phagocytes, particularly in macrophages (Fang, 2004). iNOS catalyzes the formation of NO and citrulline from L-arginine and oxygen (Stuehr, 1999). NO produced by iNOS can subsequently be converted into derivatives such as nitrogen dioxide (NO_2_), peroxynitrite (ONOO^–^), and dinitrogen trioxide (N_2_O_3_) (Fang, 2004; Stern and Zhu, 2014). Furthermore, nitrate in the diet can be reduced by commensals to nitrite, which interacts with gastric acid to result in RNS (Sobko et al., 2005; Davies et al., 2011; Tiso and Schechter, 2015) that act as antimicrobial barriers against ingested enteric pathogens (Fang, 2004). RNS can lead to the damage of cellular components, including metal centers of proteins, membrane lipids and nucleotide bases, and thereby inhibit respiration and interfere with DNA replication of pathogens (Fang, 1999). Therefore, pathogens have evolved sophisticated mechanisms to overcome nitrosative stress caused by the increased level of RNS, and the mechanisms are closely linked to their virulence (Bang et al., 2006; Richardson et al., 2006; Stern et al., 2012).

To defend against the nitrosative stress, pathogens rely on a variety of detoxifying enzymes such as NO dioxygenase, flavorubredoxin and associated oxidoreductase, cytochrome *c* nitrite reductase, *S*-nitrosoglutathione reductase, and peroxynitrite reductase (Bryk et al., 2000; Liu et al., 2001; Fang, 2004; Karlinsey et al., 2012). Among these, multidomain NO dioxygenases are a family of flavohemoglobins (Hmp) composed of the N-terminal globin domain carrying the heme-binding site and the C-terminal oxidoreductase domain containing NAD- and FAD-binding sites (Bonamore and Boffi, 2008). The NO dioxygenases typically detoxify the potentially harmful NO by oxidizing it to a less toxic metabolite NO_3_^–^ under aerobic conditions (Hausladen et al., 2001), although some of the NO dioxygenases have an additional activity to reduce NO to N_2_O in the absence of oxygen (Mills et al., 2008; Forrester and Foster, 2012). Under conditions where O_2_ is not limiting, the N-terminal ferrous-oxy (Fe^2+^-O_2_) heme reacts with NO to yield NO_3_^–^ and ferric-deoxy (Fe^3+^) heme, in which both atoms of O_2_ are incorporated into the NO_3_^–^ (Gardner A. M. et al., 2000; Forrester and Foster, 2012). The C-terminal oxidoreductase domain transfers electrons from FADH_2_ to the ferric heme to regenerate the ferrous heme and the resulting FAD^+^ is then reduced back to FADH_2_ by utilizing the reducing power of cellular NAD(P)H (Forrester and Foster, 2012).

The opportunistic human pathogen, *V. vulnificus*, is a causative agent of life-threatening septicemia and necrotizing fasciitis in individuals with predisposing conditions, such as liver damage and kidney failure (Gulig et al., 2005; Oliver, 2015). It is reasonable to assume that *V. vulnificus* has to cope with the nitrosative stress imposed by the immune system in order to survive in the host and in turn ensure developing illness. Nevertheless, no definitive analysis on the mechanisms of *V. vulnificus* in surviving under nitrosative stress and thereby exhibiting virulence has been undertaken at a molecular level until now. Accordingly, we initiated a transcriptome analysis and identified *hmpA*, a homolog of *Escherichia coli hmp* (*Echmp*), which is the most preferentially expressed in *V. vulnificus* cells exposed to NO. The biochemical and kinetic properties of *V. vulnificus* HmpA (*Vv*HmpA), the product of *VvhmpA*, were verified experimentally. Construction of the isogenic *VvhmpA* mutant and evaluation of its phenotypes provided evidence that *Vv*HmpA could contribute to the survival and thereby the pathogenesis of *V. vulnificus* during infection.

## Materials and Methods

### Strains, Plasmids, and Culture Conditions

The strains and plasmids used in this study are listed in Table 1. Unless otherwise noted, the *V. vulnificus* MO6-24/O (wild type), *VvhmpA* mutant, and *VvhmpA-*complemented strain were grown aerobically in Luria-Bertani (LB) medium supplemented with 2.0% (w/v) NaCl (LBS) at 30°C, and their growth was monitored spectrophotometrically at 600 nm (*A*_600_). The RAW 264.7 murine macrophage cells were obtained from Korean Cell Line Bank (Seoul, South Korea) and grown in Dulbecco’s modified Eagle’s medium (DMEM) containing 10% fetal bovine serum (FBS) and appropriate antibiotics [100 units ml^–1^ penicillin G and 100 μg ml^–1^ streptomycin (Gibco-BRL, Gaithersburg, MD)] in air supplemented with 5% CO_2_ at 37°C.

**TABLE 1:** Plasmids and bacterial strains used in this study.

**Strain or plasmid**	**Relevant characteristics^†^**	**References or source**
**Bacterial strains**		
*V. vulnificus*		
MO6-24/O	Wild type; clinical isolate; virulent	(Wright et al., 1990)
DY171	MO6-24/O with Δ*hmpA*	This study
*E. coli*		
S17-1 λ*pir, tra*	λ-*pir* lysogen; *thi pro hsdR hsdM^+^ recA* RP4-2 Tc:Mu-Km:Tn7; Tp^r^ Sm^r^; host for π-requiring plasmids; conjugal donor	(Simon et al., 1983)
BL21 (DE3)	F^–^ *ompT hsdS_B_* (r_B_^–^ m_B_^–^) *gal dcm* (DE3)	Laboratory collection
**Plasmids**		
pDM4	R6K γ *ori sacB*; suicide vector; *oriT* of RP4; Cm^r^	(Milton et al., 1996)
pDY1618	pDM4 with Δ*hmpA*	This study
pJH0311	Broad-host-range vector; Ap^r^, Cm^r^	(Goo et al., 2006)
pDY1701	pJH0311 with *VvhmpA*; Ap^r^, Cm^r^	This study
pPROEX HTa	His_6_–tag fusion protein expression vector; Ap^r^	Invitrogen
pDY1703	pPROEX HTa with *VvhmpA*; Ap^r^, Cm^r^	This study
pET-21c(+)	Optional C-terminal His_6_–tag fusion protein expression vector; Km^r^	Invitrogen
pSH1701	pET-21c(+) with *VvhmpA*; Km^r^	This study

*^†^Tp^*r*^, trimethoprim-resistant; Sm^*r*^, streptomycin-resistant; Cm^*r*^, chloramphenicol-resistant; Ap^*r*^, ampicillin-resistant; Km^*r*^, kanamycin-resistant.*

### RNA Purification and Transcriptome Analysis

For transcriptome analysis, total RNAs were isolated from biological duplicates of *V. vulnificus* MO6-24/O, grown aerobically to *A*_600_ of 0.5 in M9 minimal media supplemented with 0.4% (w/v) glucose (M9G) and exposed to different types of NO donors for 10 min. As NO donors, excess amounts of either NO-releasing poly(lactic-*co*-glycolic acid)-polyethylenimine nanoparticles (NO/PPNPs) (releasing NO with *t*_1__/__2_ = 24 h at 37°C) or Spermine NONOate (releasing NO with *t*_1__/__2_ = 39 min at 37°C, Cayman Chemical, Ann Arbor, MI) were used (Bang et al., 2006; Nurhasni et al., 2015). The RNAs were further purified by removing DNA using TURBO DNase (Ambion, Austin, TX), and mRNA was selectively enriched by depleting rRNA using a Ribo-Zero rRNA removal kit (Epicentre, Madison, WI) according to the manufacturer’s procedure.

The cDNA libraries were constructed using a TruSeq Stranded mRNA Sample Prep kit (Illumina, San Diego, CA). Strand-specific paired-end 100-bp sequences were read from each cDNA library using HiSeq 2500 (Illumina) as described previously (Kim et al., 2018). The raw sequencing reads were analyzed using CLC Genomics Workbench 5.5.1 (CLC Bio, Aarhus, Denmark) and mapped on to the *V. vulnificus* MO6-24/O genome sequence (GenBank^TM^ accession numbers: CP002469 and CP002470). The expression level of each gene was defined using the number of fragments per kilobase of transcript per million mapped reads (FPKM) (Conesa et al., 2016). Quantile-normalized FPKM values were then statistically analyzed by *t*-tests to identify the genes expressed differentially (fold change ≥2; *p* value of <0.05) in the *V. vulnificus* exposed to NO/PPNPs.

### qRT-PCR

One microgram of the total RNA was used to synthesize cDNA with the iScript^TM^ cDNA synthesis kit (Bio-Rad, Hercules, CA), and real-time PCR amplification of the cDNA was performed by using the Chromo 4 real-time PCR detection system (Bio-Rad) with pairs of specific primers (Supplementary Table S1) as described previously (Jang et al., 2017). Relative expression levels of the *VvhmpA* mRNA in the same amounts of total RNA were calculated by using the 16S rRNA expression level as the internal reference for normalization (Jang et al., 2017). All qRT-PCR was conducted in biological triplicates.

### Generation and Complementation of the *VvhmpA* Mutant

The *VvhmpA* gene was inactivated *in vitro* by deletion of the *VvhmpA* ORF (660-bp of 1,185-bp) using the PCR-mediated linker-scanning mutation method as described previously (Kim et al., 2014). Briefly, two pairs of primers HMPA01-F and -R (for amplification of the 5′ amplicon) and HMPA02-F and -R (for amplification of the 3′ amplicon) were designed and used (Supplementary Table S1). The *VvhmpA* gene with the 660-bp deletion was amplified by PCR using the mixture of both amplicons as the template and HMPA01-F and HMPA02-R as primers. The resulting Δ*hmpA* was ligated into *Spe*I-*Sph*I-digested pDM4 to form pDY1618 (Table 1). *E. coli* S17-1 λ*pir, tra* (Simon et al., 1983) strain containing pDY1618 was used as a conjugal donor to *V. vulnificus* MO6-24/O to generate the *VvhmpA* mutant, DY171 (Table 1). The conjugation and isolation of the transconjugant were conducted using the method described previously (Lim and Choi, 2014).

To complement the *VvhmpA* mutation, the upstream region and ORF of *VvhmpA* were amplified by PCR using HMPA03-F and HMPA03-R as primers (Supplementary Table S1). The amplified *VvhmpA* was cloned into the broad-host-range vector pJH0311 (Goo et al., 2006) to create pDY1701 (Table 1). Either pJH0311 or pDY1701 was transferred into DY171 by conjugation as described above.

### Western Blot Analysis

The ORF of *VvhmpA* was amplified by PCR using a pair of primers, HMPA04-F and -R (Supplementary Table S1), digested with *Nco*I and *Sal*I, and then ligated into pPROEX HTa (Invitrogen, Carlsbad, CA) to result in pDY1703 (Table 1). The His_6_-tagged *Vv*HmpA was expressed in *E. coli* BL21 (DE3) containing pDY1703 and purified by using affinity chromatography (Qiagen) according to the manufacturer’s procedure, and used to raise rabbit anti-*Vv*HmpA polyclonal antibody (AbFrontier, Seoul, South Korea) (Jang et al., 2017).

For Western blot analysis, *V. vulnificus* MO6-24/O grown to *A*_600_ of 0.5 in M9G was exposed either to Spermine NONOate as described above or to M9G (negative control) and then harvested to isolate total cellular proteins. To detect *Vv*HmpA, the total cellular proteins (20 μg) were resolved on SDS-PAGE under reducing conditions and immunoblotted using the rabbit anti-*Vv*HmpA antibody as described previously (Jang et al., 2017). A mouse antibody to *E. coli* DnaK was purchased (Enzo Life Sciences, Farmingdale, NY) and used to detect *V. vulnificus* DnaK (*Vv*DnaK, a loading control) (Jang et al., 2017).

### Purification of *Vv*HmpA and Absorption Spectra of the Reduced, NO-Bound, and Oxidized *Vv*HmpA

The *VvhmpA* ORF was amplified by PCR using a pair of primers, HMPA05-F and -R (Supplementary Table S1), digested with *Nde*I and *Xho*I, and then ligated into pET-21c(+) (Invitrogen) to result in pSH1701 (Table 1). The non-His_6_-tagged *Vv*HmpA was expressed in *E. coli* BL21 (DE3) containing pSH1701 and purified by using a HiTrap Q anion-exchange column (GE Healthcare, Chicago, IL) and then a HiLoad Superdex 16/60 200 column (GE Healthcare) as described previously (Ahn et al., 2018). The protein was dissolved in the buffer with 20 mM Tris–HCl (pH 8.0) and 0.25 M NaCl at 4°C until use. The purified *Vv*HmpA was quantitated using the Bradford method (Bradford, 1976).

To obtain the reduced *Vv*HmpA, the purified *Vv*HmpA (10 μM) was mixed with sodium hydrosulfite powder (1 μM, Sigma, St. Louis, MO) under anaerobic conditions (Ioannidis et al., 1992). Immediately, the residual sodium hydrosulfite was removed by using Ultracell-10K centricon (Millipore, Burlington, MA) in the anaerobic chamber with an atmosphere of 90% N_2_, 5% CO_2_, and 5% H_2_ (Coy Laboratory Products, Grass Lake, MI) (Kim et al., 2014). The reduced *Vv*HmpA (10 μM) was incubated under anaerobic conditions with 40 μM Diethylamine NONOate sodium salt hydrate (Sigma) (releasing NO with *t*_1__/__2_ = 2 min at 37°C) for 10 min to obtain the NO-bound *Vv*HmpA, or under aerobic conditions for 20 min to obtain the oxidized *Vv*HmpA. The UV–vis absorption spectra of the *Vv*HmpA proteins were recorded by using Shimadzu UV-1800 UV/VIS spectrophotometer (Shimadzu, Kyoto, Japan) at room temperature.

### Reconstitution of *Vv*HmpA With Heme and FAD and Quantitation of the Cofactors

To reconstitute *Vv*HmpA with heme *in vitro*, 10 μM of the purified *Vv*HmpA was gradually mixed with stoichiometric excess of hemin (1 mM in 0.01 M NaOH), and then incubated for 40 min at 4°C (Gardner A. M. et al., 2000). After the residual hemin aggregates were removed by centrifugation, the *Vv*HmpA saturated with heme was further purified using a HiPrep^TM^ 26/10 column (GE Healthcare). Similarly, 10 μM of the purified *Vv*HmpA was incubated with 80 μM of FAD for an hour at room temperature, and then residual FAD was removed from the resulting reconstituted *Vv*HmpA using Ultracell-10K centricon (Fang et al., 2017). The contents of the heme and FAD in *Vv*HmpA both before and after the reconstitution with each of the cofactor were determined using the pyridine hemochromagen assay (Barr and Guo, 2015) and FAD fluorometric assay (Hirano and Namihira, 2017), respectively, as described previously.

### Kinetic Analysis

Fresh NO stock solution was prepared daily as described previously (Poole et al., 1996) with modifications. Briefly, NO gas was formed by reacting 0.25 g sodium nitrite with 5 ml acidic ferrous sulfate solution, and purified by passing through 1 M NaOH solution and then degassed distilled water. The purified NO gas was collected and then dissolved in degassed distilled water to form the NO stock solution of approximately 500 μM, which was serially diluted to appropriate concentrations immediately before use.

To determine the kinetic properties of *Vv*HmpA, 10 μl of the distilled water containing varying concentrations of NO and 10 μl of the purified and heme-reconstituted *Vv*HmpA (1 μM) were delivered via separate syringes to 980 μl of the reaction buffer (50 mM potassium phosphate (pH 7.8) with 100 μM EDTA, 1 μM FAD and 100 μM NADH), that is pre-incubated at either 37 or 30°C (Gardner A. M. et al., 2000). The initial rates of NO decomposition were obtained by measuring the residual NO in the reaction mixture amperometrically using an ISO-NOP electrode (World Precision Instruments, Sarasota, FL) and plotted against the concentrations of NO. *K*_M_ was determined as the NO concentration at which *Vv*HmpA decomposes NO at one half rate of its *V*_max_ (Smagghe et al., 2008) and the *k*_cat_ was determined through the Lineweaver–Burk plotting (Chapman and Reid, 1999). Presuming only *Vv*HmpA molecules containing heme are active, the *k*_cat_ was expressed relative to heme.

### NO-Decomposition Activity and Survival of *V. vulnificus*

The *V. vulnificus* strains grown to *A*_600_ of 0.5 in M9G were pre-incubated with 50 μM Spermine NONOate for 30 min to induce *Vv*HmpA. The *V. vulnificus* cells were harvested with centrifugation, washed twice using PBS, and resuspended with 10 ml PBS. The PROLI NONOate (releasing NO with *t*_1__/__2_ = 1.8 s at 37°C, Cayman Chemical) was administered to the resuspended *V. vulnificus* cells to achieve 2 μM NO at a final concentration, and their NO-decomposition activities were determined by measuring the residual NO using the ISO-NOP electrode.

To examine the effect of *Vv*HmpA on the survival of *V. vulnificus* under nitrosative stress, equal numbers (approximately 10^7^ cells ml^–1^) of the *V. vulnificus* strains were used to inoculate the M9G containing 0.15 mg ml^–1^ NO/PPNPs at a final concentration. The resulting cultures were further incubated aerobically with shaking and the viable cells were counted at time intervals.

### Survival and Cytotoxicity of *V. vulnificus* Infecting Immune Cells

The macrophage RAW 264.7 cells were resuspended in fresh DMEM containing 500 ng ml^–1^ *E. coli* O111:B4 lipopolysaccharide (Sigma) and 1 mM L-arginine (Sigma) to induce NO production (Walker et al., 1997) either with or without 500 μM L-N^G^-monomethyl arginine citrate (L-NMMA, Sigma), which is a known NO synthase inhibitor (Nathan and Hibbs, 1991). The RAW 264.7 cells were seeded into 24-well culture dishes at a concentration of 5 × 10^5^ cells per well, and infected with the *V. vulnificus* strains at a multiplicity of infection (MOI) of 1. To determine the survival of the *V. vulnificus* strains directly affected by the NO from the RAW 264.7 cells, the culture dishes were washed two times to remove bacteria non-adherent to the macrophages as described previously (Lim and Choi, 2014). Following the last wash, the RAW 264.7 cells were broken with 0.1% Triton X-100 treatment for 20 min, and the recovered bacterial cells were enumerated as cfu per well (Lim and Choi, 2014). The numbers of live RAW 264.7 cells were determined at each time point using the LIVE/DEAD Viability/Cytotoxicity Kit for mammalian cells (Invitrogen) following manufacturer’s procedure, and used to result in numbers of bacteria per macrophage at each time point. The cytotoxicity of the *V. vulnificus* strains was determined by measuring the lactate dehydrogenase (LDH) activity released into the supernatant as described previously (Lim and Choi, 2014), and expressed using the LDH activity released from the RAW 264.7 cells completely lysed by 1.5% Triton X-100 (Sigma) as 100%. Statistical significance was determined by the Student’s *t* test.

### Mouse Lethality Assay

The *V. vulnificus* strains grown to *A*_600_ of 0.5 were harvested and resuspended in PBS to 1.0 × 10^7^ cfu ml^–1^. To determine mouse lethality, 100 μl of the suspension of either the wild type or *VvhmpA* mutant was used for intraperitoneal infection of the 7-week-old Institute of Cancer Research (ICR) female mice (specific-pathogen-free, Seoul National University) (*n* = 5 for each infection). The infection experiments were performed three times with the mice (total *n* = 15) to ensure reproducibility and percentages of the mice survival were recorded for 24 h. Statistical significance was determined by the log-rank test. All manipulations for mouse lethality assay were approved by the Animal Care and Use Committee at Seoul National University.

### Data Analyses and Transcriptome Data Accession Number

Averages and standard deviations (S.D.) were calculated from at least three independent experiments. Statistical analyses were performed using GraphPad Prism 7.0 (GraphPad Software). All raw transcriptome data were deposited in Sequence Read Archive (SRA)^1^ under accession number PRJNA513463^2^.

## Results

### Effects of NO on *V. vulnificus* Transcriptome

**FIGURE 1:**
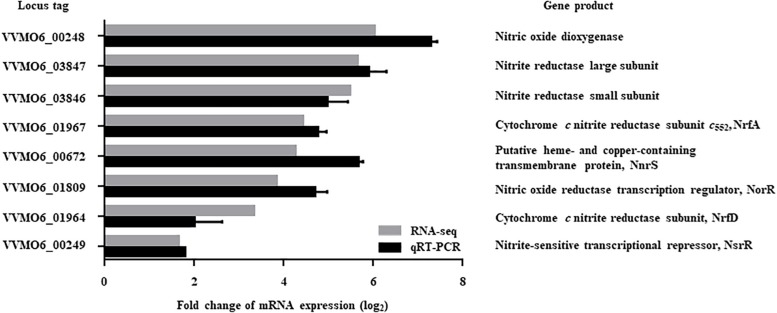
Genes upregulated by NO exposure and possibly involved in nitrosative stress defense. Among the NO-upregulated genes (fold change ≥2; *p* value of <0.05) identified by transcriptome analysis, 8 genes potentially involved in nitrosative stress defense were selected and their upregulation was confirmed by qRT-PCR. Each column represents the mRNA expression level of *V. vulnificus* MO6-24/O exposed to NO/PPNPs relative to that exposed to PPNPs (negative control). *Error bars* represent the S.D. Locus tags are based on the *V. vulnificus* MO6-24/O genome sequence (GenBank^TM^ accession numbers: CP002469 and CP002470) and the products of the genes are presented on the right. *NO/PPNPs*, NO-releasing poly(lactic-*co*-glycolic acid)-polyethylenimine nanoparticles; *PPNPs*, poly(lactic-*co*-glycolic acid)-polyethylenimine nanoparticles.

The *V. vulnificus* MO6-24/O cells exposed to NO/PPNPs (Nurhasni et al., 2015) were harvested and their transcriptomes were analyzed. Transcriptome analysis revealed 551 genes that were differentially expressed (fold change ≥2; *p* value <0.05) upon exposure to NO; 320 genes were upregulated and 231 genes were downregulated (data were deposited in SRA, see text footnote 1, under accession number PRJNA513463, Supplementary Tables S2, S3). Among the genes upregulated upon exposure to NO, 8 genes potentially involved in nitrosative stress defense were selected (Figure 1). Six of the genes are predicted to encode proteins involved in the defense against nitrosative stress: NO dioxygenase, nitrite reductase large subunit, nitrite reductase small subunit, NrfA (cytochrome *c* nitrite reductase subunit *c*_552_), NnrS (putative heme- and copper-containing transmembrane protein), and NrfD (cytochrome *c* nitrite reductase subunit). The other two genes are predicted to encode transcriptional regulators that control the expression of the genes involved in nitrosative stress defense: NorR (NO reductase transcription regulator) and NsrR (nitrite-sensitive transcriptional repressor).

**FIGURE 2:**
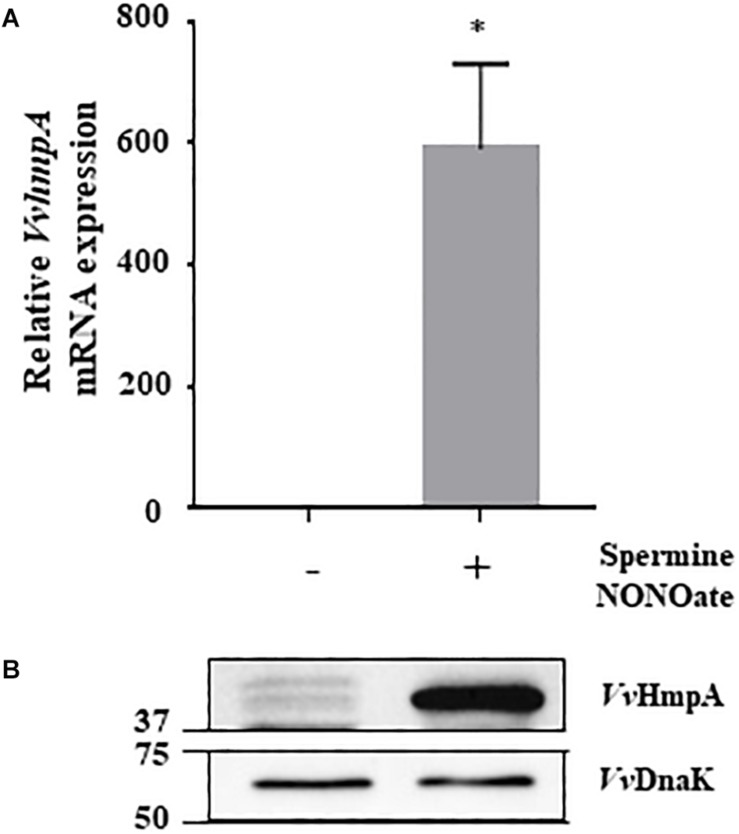
Expression of *VvhmpA* under nitrosative stress. RNAs and proteins were extracted from *V. vulnificus* MO6-24/O exposed either to Spermine NONOate or M9G (negative control). **(A)** The *VvhmpA* mRNA levels were determined by qRT-PCR analyses, and expressed using the *VvhmpA* mRNA level of the culture unexposed to NO as 1. *Error bars* represent the S.D. ^∗^*p* < 0.05 relative to the culture unexposed to NO. **(B)** Total proteins of the cultures were resolved on reducing SDS-PAGE, and *Vv*HmpA and *Vv*DnaK were immunoblotted using the rabbit anti-*Vv*HmpA antibody and the mouse anti-DnaK antibody, respectively. The protein size markers (Bio-Rad) on the left are in kilodaltons.

Expression of the 8 genes in *V. vulnificus* exposed to NO/PPNPs was reevaluated by using quantitative real-time PCR (qRT-PCR) analyses, further confirming that NO exposure induced transcription of the genes (Figure 1). Since the expression of the VVMO6_00248 gene, predicted to encode an NO dioxygenase, increased the most upon exposure to NO (Figure 1), the gene was selected for further study. To verify that the induction of the VVMO6_00248 gene is not confined to a specific NO donor, expression of the gene upon exposure of *V. vulnificus* to Spermine NONOate as an alternate NO donor was determined. As shown in Figure 2, exposure to the Spermine NONOate increased the levels of the gene product as well as the transcript of VVMO6_00248 as determined by Western blot analysis and qRT-PCR, respectively. The combined results confirmed that the expression of the VVMO6_00248 gene in *V. vulnificus* is induced upon exposure to NO.

### Identification and Sequence Analysis of *Vv*HmpA

**FIGURE 3:**
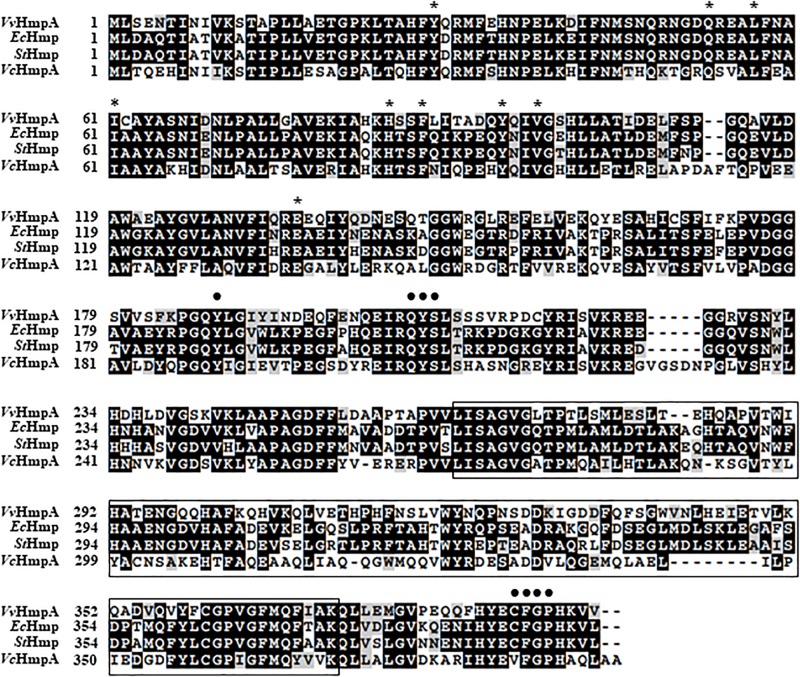
Sequence analysis of *V. vulnificus* HmpA (*Vv*HmpA), *E. coli* Hmp (*Ec*Hmp), *S.* Typhimurium Hmp (*St*Hmp), and *V. cholerae* HmpA (*Vc*HmpA). The amino acid sequences retrieved from the NCBI protein database (accession numbers: WP_013570994.1 for *Vv*HmpA, NP_417047.1 for *Ec*Hmp, WP_000883146.1 for *St*Hmp, and WP_000957477.1 for *Vc*HmpA) were aligned using the Clustal Omega program. Identical (*black boxes*), conserved (*gray boxes*), and missing (*dashes*) sequences are indicated. The conserved amino acid residues potentially involved in the binding of heme and FAD are indicated *above* the amino acid sequences by *asterisks* and *dots*, respectively. The putative NAD-binding domain is *boxed* by *a black line*.

The amino acid sequence deduced from the nucleotide sequence of the VVMO6_00248 gene revealed a putative protein, composed of 394 amino acids with a theoretical molecular mass of 44.3 kDa and a pI of 5.21. The deduced amino acid sequence of VVMO6_00248 was 62%, 61%, and 51% identical to known NO dioxygenases such as *E. coli* Hmp (*Ec*Hmp), *Salmonella enterica* serovar Typhimurium Hmp (*St*Hmp), and *Vibrio cholerae* HmpA (*Vc*HmpA) (Figure 3), respectively. These findings led us to assume that the protein is an NO dioxygenase of *V. vulnificus* and thus named it *Vv*HmpA. Amino acid sequence analysis of *Vv*HmpA further revealed that the protein possesses the highly conserved heme-binding domain of known NO dioxygenases (Bonamore and Boffi, 2008) (Figure 3). Moreover, *Vv*HmpA contains the putative NAD- and FAD-binding domains, which are also conserved in NO dioxygenases (Bonamore and Boffi, 2008) (Figure 3). The combined results proposed that *Vv*HmpA is a multidomain NO dioxygenase that possibly contains heme and FAD as its cofactors.

### *Vv*HmpA Is a Flavohemoglobin

**FIGURE 4:**
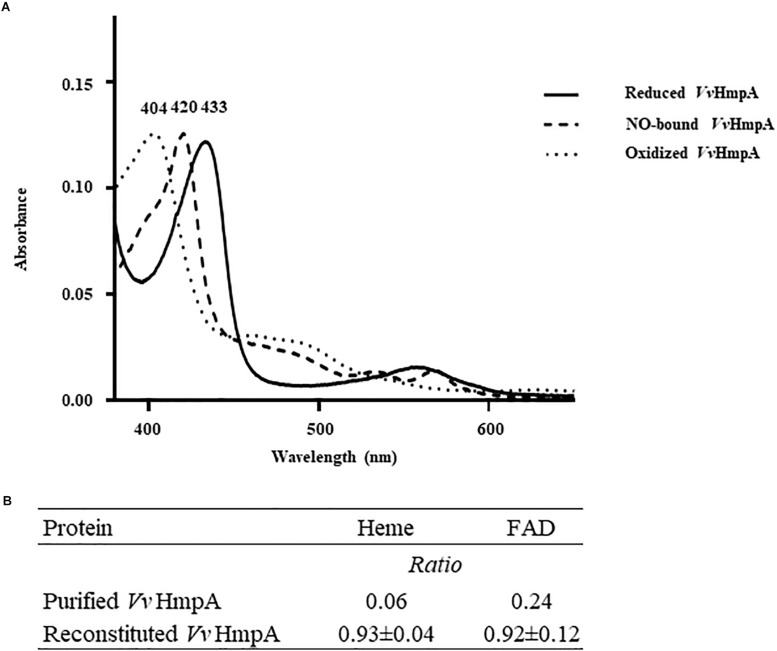
Absorption spectra and cofactors of *Vv*HmpA. **(A)** The absorption spectra of the purified *Vv*HmpA in the reduced (*solid line*), NO-bound (*dashed line*), and oxidized (*spotted line*) states were measured using a UV–vis spectrophotometer. The numbers above the peaks indicate the wavelengths of the observed Soret peaks. **(B)** The ratios of heme and FAD per one molecule of purified and reconstituted *Vv*HmpA.

Absorption spectra of *Vv*HmpA in solution were characterized to reveal physical properties of the protein. A narrow intense band centered at approximately 433 nm, referred to as the Soret band and characteristic of heme proteins (Anderson and Robertson, 1995), was observed in the absorption spectrum of the reduced *Vv*HmpA (Figure 4A). The 433 nm Soret peak of the reduced *Vv*HmpA was altered to the 420 and 404 nm Soret peaks of the NO-bound and oxidized *Vv*HmpAs, respectively (Figure 4A). These Soret peak alterations most probably resulted from the changes in the physical state of iron in the heme protein, as demonstrated previously with a heme protein *Ec*Hmp which showed Soret peaks at 431.5, 419, and 403.5 nm in its reduced, NO-bound, and oxidized states, respectively (Ioannidis et al., 1992; Anderson and Robertson, 1995; Gardner A. M. et al., 2000). Therefore, it is reasonable to propose that *Vv*HmpA is also a heme protein, in which iron can exist in either reduced, NO-bound, or oxidized state.

Measurement of heme content revealed that the overexpressed and purified *Vv*HmpA contains 0.06 molecules of heme per *Vv*HmpA monomer (Figure 4B). It was possible that the amount of heme provided in the condition used for the overexpression and purification of the *Vv*HmpA protein is not sufficient to saturate the protein. To examine the possibility, the purified *Vv*HmpA was saturated with excess hemin *in vitro* (Gardner A. M. et al., 2000), and then the content of heme in the reconstituted *Vv*HmpA was determined. Following the saturation, the content of heme in the *Vv*HmpA increased to 0.93 ± 0.04 molecules of heme per *Vv*HmpA monomer (Figure 4B). These results suggested that holo-*Vv*HmpA contains approximately one molecule of heme per protein monomer.

FAD fluorometric assay was carried out in order to determine the FAD content of the overexpressed and purified *Vv*HmpA. Prior to saturation with excess FAD *in vitro*, the purified *Vv*HmpA contained 0.24 molecules of FAD per *Vv*HmpA monomer (Figure 4B). After saturation with excess FAD *in vitro*, the content of FAD in the reconstituted *Vv*HmpA increased to 0.92 ± 0.12 molecules of FAD per *Vv*HmpA monomer (Figure 4B), suggesting that the holo-*Vv*HmpA contains approximately one molecule of FAD per protein monomer. The combined results proposed that the holo-*Vv*HmpA protein is a flavohemoglobin that contains equimolar amounts of heme and FAD as cofactors.

### Kinetic Properties of *Vv*HmpA for NO Decomposition

**FIGURE 5:**
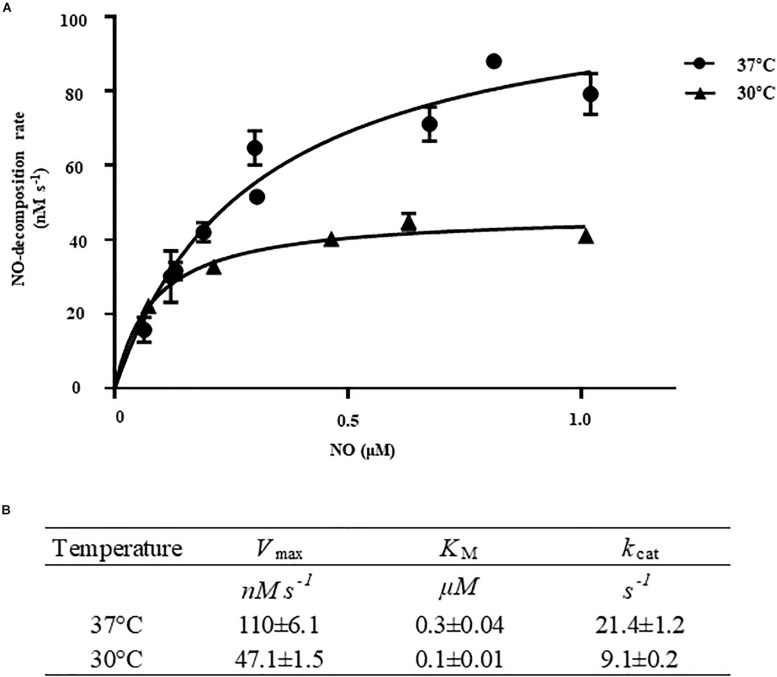
Kinetic analysis of *Vv*HmpA. **(A)** The *Vv*HmpA protein and various concentrations of NO were delivered into the reaction buffer. The initial rates of NO decomposition were determined by measuring the residual NO in the reaction mixture and plotted against the corresponding initial concentrations of NO. *Error bars* represent the S.D. **(B)** *V*_max_, *K*_M_, and *k*_cat_ values for NO were determined by fitting the curve **(A)** to a classical Michaelis–Menten enzyme kinetic equation. The turnover rate (*k*_cat_) is expressed relative to heme.

To determine the kinetic properties of *Vv*HmpA, the initial rates of NO decomposition were measured at different concentrations of NO. At the concentrations of NO exceeding 0.15 μM, which are most probably encountered in the human immune system (Toledo and Augusto, 2012), the NO-decomposition rate of *Vv*HmpA was higher at 37°C rather than at 30°C (Figure 5A). The *V*_max_ of *Vv*HmpA for NO, obtained from the Michaelis–Menten plot, was approximately 110.1 ± 6.1 nM s^–1^ at 37°C and the NO-decomposition rate at the concentration of 1 μM NO was approximately 80% of the *V*_max_ value (Figure 5). In contrast, the *V*_max_ of *Vv*HmpA for NO at 30°C was about 47 ± 1.5 nM s^–1^ and the NO-decomposition rate at the concentration of 1 μM NO was approximately 90% of the *V*_max_ value (Figure 5). The *K*_M_ values of *Vv*HmpA for NO were 0.3 ± 0.04 μM and 0.1 ± 0.01 μM at 37 and 30°C, respectively (Figure 5B). The *k*_cat_ values of *Vv*HmpA for NO were 21.4 ± 1.2 s^–1^ and 9.1 ± 0.2 s^–1^ at 37 and 30°C, respectively (Figure 5B). Considering that *V. vulnificus* is a pathogen infecting humans with the body temperature of 37°C, the results indicated that *Vv*HmpA is more efficient at decomposing high levels of toxic NO in the host than in the natural environment.

### *Vv*HmpA Is Essential for the Survival of *V. vulnificus* Under Nitrosative Stress *in vitro*

**FIGURE 6:**
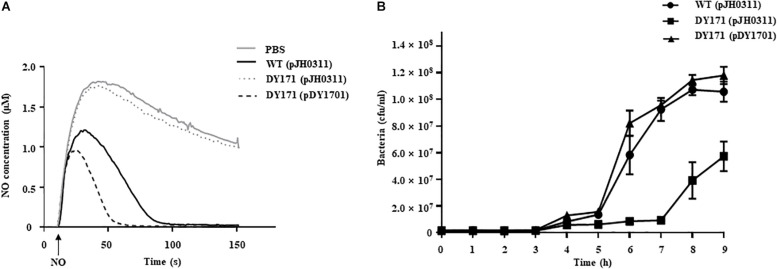
NO decomposition and survival of the *V. vulnificus* strains under nitrosative stress. **(A)** The *V. vulnificus* strains were pre-exposed to Spermine NONOate to induce *Vv*HmpA, and then 2 μM of NO was administered to the strains at the time designated by *an arrow*. The residual NO in the mixtures was measured to determine the NO decomposition. **(B)** Survival of the *V. vulnificus* strains exposed to excess NO was monitored by counting viable cells at time intervals. *Error bars* represent the S.D. *PBS*, control; *WT (pJH0311)*, wild type; *DY171 (pJH0311), VvhmpA* mutant; *DY171 (pDY1701), VvhmpA-*complemented strain.

To evaluate the role of *Vv*HmpA in *V. vulnificus* encountering nitrosative stress, the NO-decomposition activities of the *V. vulnificus* strains were compared (Figure 6A). When NO was administered to the *V. vulnificus* wild-type culture, the NO concentration decreased rapidly in the culture and the residual NO was not detectable after 100 s, indicating that the *V. vulnificus* wild type effectively decomposes NO *in vitro*. In contrast, the rate at which NO levels decrease in the *VvhmpA* mutant culture was much slower than that in the wild-type culture (Figure 6A). The rate at which NO levels decrease in the *VvhmpA* mutant culture was close to that in the medium without bacterial inoculation (control, PBS), indicating that the NO-decomposition activity of the *VvhmpA* mutant was significantly impaired (Figure 6A). By complementation of the *VvhmpA* gene, the impaired NO-decomposition activity of the *VvhmpA* mutant was restored to a level even higher than that of the wild type (Figure 6A). The results suggested that the NO-decomposition activity of *V. vulnificus* is mostly dependent on *Vv*HmpA *in vitro*.

To examine the effects of *Vv*HmpA on the survival of *V. vulnificus* under nitrosative stress *in vitro*, the growth of *V. vulnificus* strains in M9G was compared in the presence of NO (Figure 6B). The growth of the *VvhmpA* mutant was significantly delayed compared with that of the wild type. That is, the *VvhmpA* mutant did not enter the exponential phase until 7 h post-inoculation, which is delayed for approximately 2 h compared with the wild type. The delayed growth of the *VvhmpA* mutant was restored in the *VvhmpA-*complemented strain (Figure 6B), indicating that *Vv*HmpA is able to effectively decompose toxic NO to the level at which *V. vulnificus* can manage to grow. The combined results led us to conclude that *Vv*HmpA detoxifies NO effectively *in vitro*, which in turn ensures survival of the pathogen under nitrosative stress.

### *Vv*HmpA Is Essential for the Virulence of *V. vulnificus ex vivo*

**FIGURE 7:**
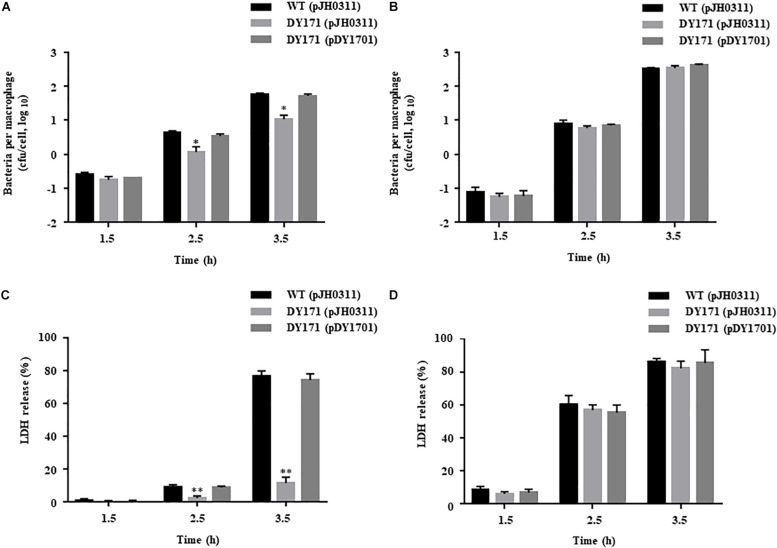
Survival and cytotoxicity of the *V. vulnificus* strains infecting the RAW 264.7 cells. The NO-producing RAW 264.7 cells were infected with the *V. vulnificus* strains at the MOI of 1 for various incubation times in the absence **(A,C)** or presence **(B,D)** of the NO synthase inhibitor, L-NMMA. **(A,B)** The *V. vulnificus* cells adherent to the RAW 264.7 cells were enumerated in cfu per macrophage at each time point after infection. **(C,D)** The cytotoxicity was expressed using the total LDH activity of the RAW 264.7 cells completely lysed by 1.5% Triton X-100 as 100%. *Error bars* represent the S.D. ^∗^*p* < 0.05 and ^∗∗^*p* < 0.005 relative to groups infected with the wild type at each incubation time. *WT (pJH0311)*, wild type; *DY171 (pJH0311), VvhmpA* mutant; *DY171 (pDY1701)*, *VvhmpA-*complemented strain.

To examine the survival of *V. vulnificus* strains in the presence of NO-producing murine macrophage RAW 264.7 cells, the numbers of the *V. vulnificus* cells adherent to the RAW 264.7 cells were measured. The numbers of the *VvhmpA* mutant per macrophage were significantly lower than those of the wild type and *VvhmpA*-complemented strain (Figure 7A), indicating that *Vv*HmpA is required for *V. vulnificus* to survive in proximity to the NO-producing macrophages. Accordingly, when the NO production from the RAW 264.7 cells was inhibited by the NO synthase inhibitor L-NMMA, the numbers of the wild type, *VvhmpA* mutant, and *VvhmpA-*complemented strain adherent to the RAW 264.7 cells were comparable (Figure 7B). The results indicated that *Vv*HmpA is crucial for *V. vulnificus* to overcome nitrosative stress imposed by the host immune cells and thereby survive during infection.

To examine the role of *Vv*HmpA in the virulence of *V. vulnificus ex vivo*, the activities of LDH released from RAW 264.7 cells infected with the *V. vulnificus* strains were determined. As shown in Figure 7C, the *VvhmpA* mutant exhibited significantly lower LDH-releasing activity compared with those of the wild type or the *VvhmpA*-complemented strain. In contrast, when the NO production from the RAW 264.7 cells was inhibited by L-NMMA, the wild type, *VvhmpA* mutant, and *VvhmpA*-complemented strain exhibited comparable levels of LDH-releasing activity (Figure 7D). Notably, the expression levels of the well-known cytotoxic virulence factors RtxA and VvhA in the wild type, *VvhmpA* mutant, and *VvhmpA*-complemented strain were not significantly different (Supplementary Figure S1). These results combined indicated that *Vv*HmpA contributes to the virulence of *V. vulnificus* by coping with NO released from the host immune cells.

### *Vv*HmpA Is Important for the Pathogenesis of *V. vulnificus in vivo*

**FIGURE 8:**
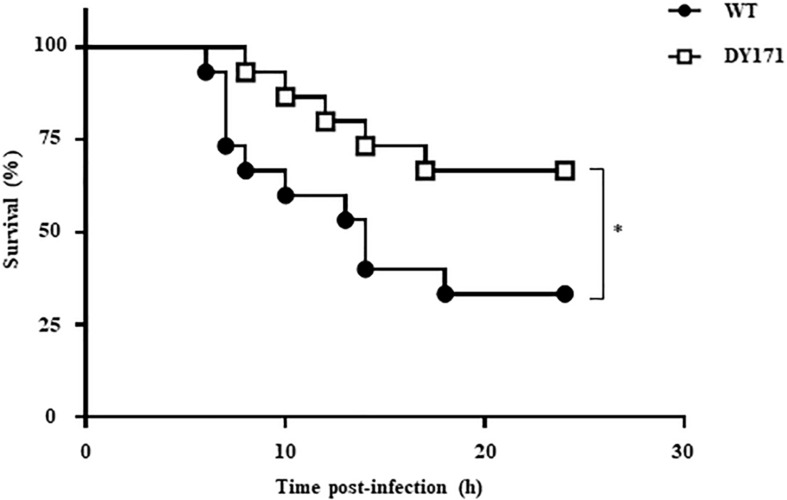
Mouse lethality of the *V. vulnificus* strains. Groups (*n* = 15) of 7-week-old specific pathogen-free female ICR mice were intraperitoneally infected with either the wild type or the *VvhmpA* mutant of *V. vulnificus* at doses of 1.0 × 10^6^ cfu. Mouse survival percentage was monitored for 24 h. ^∗^*p* < 0.05; *WT*, wild type; *DY171, VvhmpA* mutant.

The importance of *Vv*HmpA in the *V. vulnificus* pathogenesis was further investigated in a mouse model. As shown in Figure 8, the survival time of mice infected with the *VvhmpA* mutant was consistently prolonged (*p* value of 0.0420, log-rank test) compared with that of mice infected with the wild type. At 24 h post-infection, percentages of mice that survived after challenge with the *VvhmpA* mutant or the wild type were 66.6 and 33.3%, respectively, indicating that the deletion of *VvhmpA* attenuated the virulence of *V. vulnificus* in mice. Taken together, the combined results suggest that *Vv*HmpA is important for NO decomposition, survival adjacent to NO-producing immune cells, and thereby the pathogenesis of *V. vulnificus*.

## Discussion

In order to establish infection successfully, pathogens need to overcome nitrosative stresses that originate from the host immune system (Fang, 2004). In addition to the 8 genes, whose expression was further confirmed by qRT-PCR analysis in the present study (Figures 1, 2), transcriptome analysis of *V. vulnificus* identified 15 more genes that are induced upon exposure to NO and potentially involved in nitrosative stress defense (Supplementary Table S2). Among the 23 genes, *VvhmpA* was the most greatly induced and thus its gene product *Vv*HmpA was selected and further analyzed at amino acid sequence levels. The deduced amino acid sequence revealed that *Vv*HmpA is a multidomain NO dioxygenase consisting of the N-terminal globin domain with the heme-binding site and the C-terminal oxidoreductase domain with NAD- and FAD-binding sites (Figure 3). As well appreciated in a multidomain *Ec*Hmp, the N-terminal ferrous-oxy heme reacts with NO to yield NO_3_^–^ and ferric-deoxy heme, and then the C-terminal domain transfers electrons from NAD(P)H to the ferric heme via FAD (Gardner A. M. et al., 2000). This endogenous electron transfer could allow the efficient regeneration of the ferrous heme, which is ready for another catalytic cycle of NO decomposition. In contrast, single-domain NO dioxygenases found in some pathogens such as *Campylobacter jejuni* lack the C-terminal domain and rely on an exogenous redox partner(s) to regenerate ferrous heme, resulting in less efficient NO decomposition (Shepherd et al., 2011). Therefore, *V. vulnificus* seems to adopt the multidomain *Vv*HmpA to efficiently overcome nitrosative stress during infection.

Biochemical and kinetic analyses of *Ec*Hmp proposed two possible reaction mechanisms to initiate NO decomposition, namely dioxygenation and nitrosylation (Gardner A. M. et al., 2000; Hausladen et al., 2001; Forrester and Foster, 2012). Dioxygenation begins with O_2_ binding to the ferrous heme to result in ferrous-oxy heme, most probably under conditions where O_2_ is not limiting. The ferrous-oxy heme reacts with NO to form a transient Fe-ONOO^–^ intermediate which releases NO_3_^–^ as described above. In contrast, nitrosylation involves initial reaction of the ferrous heme with NO to form a ferrous-nitrosyl heme, converting transiently to a ferric-nitrosyl heme which in turn reacts with O_2_ to release NO_3_^–^ under O_2_ limiting conditions. Regardless of which mechanistic pathway predominates, they both result in rapid enzymatic turnover of NO to NO_3_^–^. Although the exact mechanisms of *Vv*HmpA detoxifying NO have not been yet clarified, the similarities found in the amino acid sequences and absorption spectra of *Ec*Hmp and *Vv*HmpA (Figures 3, 4A) led us to postulate that *Vv*HmpA also could convert NO to NO_3_^–^ through one of the two pathways depending on O_2_ availability. Supporting this postulation, the kinetic values of *Vv*HmpA for NO decomposition under aerobic conditions (Figure 5) are also comparable to those of *Ec*Hmp, in which the *k*_cat_ and *K*_M_ values are 10-670 s^–1^ and 0.25 μM for NO, respectively (Gardner P. R. et al., 2000; Gardner, 2005).

After infection, pathogenic bacteria probably encounter increased levels of NO due to the expression of iNOS in cells of the immune system and must overcome the nitrosative stress for successful pathogenesis (Bang et al., 2006; Richardson et al., 2006; Stern et al., 2012). The NO concentration in humans and experimental animals increases to the micromolar range under infectious and inflammatory conditions (Toledo and Augusto, 2012). It is noteworthy that the *K*_M_ and *k*_cat_ values of *Vv*HmpA for NO at 37°C, the temperature to which *V. vulnificus* is inevitably exposed in the host, are greater than those at 30°C (Figure 5). This kinetic property indicates that *Vv*HmpA expressed in the host may be more optimized for acting on the large amounts of NO and decomposing them rapidly to a safe level during infection, before the increased nitrosative stress impairs cellular components of *V. vulnificus*. It should be noted, however, that *Vv*HmpA may not be the sole enzyme that protects *V. vulnificus* from NO as the *VvhmpA* mutation reduced the growth and survival of the pathogen significantly but not completely (Figures 6B, 7A). Indeed, analysis of the *V. vulnificus* transcriptome upon exposure to NO showed upregulation of the genes encoding a number of NO detoxifying enzymes in addition to NO dioxygenase (Figure 1 and Supplementary Table S2) (Poole, 2005). Nevertheless, it is reasonable to hypothesize that *Vv*HmpA could provide an evolutionary advantage for *V. vulnificus* to survive in the host rather than in nature, where lower concentration of NO may be occasionally encountered. Supporting this hypothesis, loss of *Vv*HmpA led *V. vulnificus* to show significantly reduced survival adjacent to the NO-producing macrophage cells (Figures 7A,B), cytotoxicity to the immune cells (Figures 7C,D), and possibly virulence in mice (Figure 8).

It has been reported that the Hmp proteins of miscellaneous pathogenic bacteria including *V. cholerae*, *Salmonella* Typhimurium and *Staphylococcus aureus* were responsible for NO detoxification and thus required for their survival under nitrosative stress and successful pathogenesis (Bang et al., 2006; Richardson et al., 2006; Stern et al., 2012). However, little has been known about the biochemical and kinetic properties of the Hmp proteins of the pathogens until now. In the present study, absorption spectral and biochemical analyses revealed that *Vv*HmpA is a flavohemoglobin containing equimolar amounts of heme and FAD as cofactors. Kinetic properties of *Vv*HmpA with greater *K*_M_ and *k*_cat_ values at 37°C than 30°C indicated that *Vv*HmpA is optimized to act on and decompose large amounts of NO more effectively in the host. Reduced NO-decomposition activity and growth rate of the *VvhmpA* mutant in the presence of NO, along with the attenuated virulence observed both in the cell culture and mouse models, indicated that *Vv*HmpA contributes to the survival of *V. vulnificus* under nitrosative stress, and might play an important role in the pathogenesis of the pathogen. Therefore, *Vv*HmpA could be a promising target for development of new antibacterial agents.

## Data Availability Statement

The datasets generated for this study can be found in the Sequence Read Archive – PRJNA513463; https://www.ncbi.nlm.nih.gov/sra/?term=PRJNA513463.

## Ethics Statement

The animal study was reviewed and approved by Animal Care and Use Committee at Seoul National University.

## Author Contributions

DK, EN, and SC designed the research. DK, EN, SK, JK, YJ, and JC performed the research. DK, EN, IB, N-CH, and SC wrote the manuscript. All authors made major contributions to the acquisition, analysis, and interpretation of the data.

## Conflict of Interest

The authors declare that the research was conducted in the absence of any commercial or financial relationships that could be construed as a potential conflict of interest.

## Footnotes

1 https://www.ncbi.nlm.nih.gov/sra

2 https://www.ncbi.nlm.nih.gov/sra/?term=PRJNA513463
