# Supplementary material for: Transcriptomic Identification and Biochemical Characterization of HmpA, a Nitric Oxide Dioxygenase, Essential for Pathogenesis of Vibrio vulnificus
Source: Front Microbiol. 2019 Sep 24;10:2208. doi: 10.3389/fmicb.2019.02208 (PMC6768983; doi:10.3389/fmicb.2019.02208)
Supplement: Supplementary file 1 [file Table_1.docx]

**Figure legend**

**Figure S1. Expression of toxins upon exposure to RAW 264.7 cells.** The macrophage RAW 264.7 cells were resuspended in fresh DMEM containing 500 ng ml^-1^ *E. coli* O111:B4 lipopolysaccharide (Sigma) and 1 mM L-arginine (Sigma) to induce NO production. The RAW 264.7 cells were seeded into 24-well culture dishes at a concentration of 5 × 10^5^ cells per well, and infected with the *V. vulnificus* strains at a multiplicity of infection (MOI) of 1 for 90 min. Then the *V. vulnificus* strains were harvested and their expression levels of *rtxA* and *vvhA* were determined by qRT-PCR as described in the main text’s Materials and Methods. *A,* the *rtxA* mRNA levels were determined and expressed using the *rtxA* mRNA level of the wild type as 1. *B,* the *vvhA* mRNA levels were determined and expressed using the *vvhA* mRNA level of the wild type as 1. *Error bars* represent the S.D. *WT (pJH0311)*, wild type; *DY171 (pJH0311), VvhmpA* mutant; *DY171 (pDY1701), VvhmpA-*complemented strain; *ns*, not significant.

**Table S1**

**Oligonucleotides used in this study**

| Name | Oligonucleotide sequence (5' → 3') ^†, ‡^ | Use |
| --- | --- | --- |
| For qRT-PCR | | |
| 16S-qRT-F | CGGCAGCACAGAGAAACTTG | Quantification of the 16S rRNA expression |
| 16S-qRT-R | CCGTAGGCATCATGCGGTAT |  |
| 00248-qRT-F | GGCCGAAACAGGACCAAAAC | Quantification of the VVMO6_00248 expression |
| 00248-qRT-R | TGCGAGTTTGACTTTGCTGC |  |
| 03847-qRT-F | GCAAAAGCGGTGTGGTGATT | Quantification of the VVMO6_03847 expression |
| 03847-qRT-R | CAGCCATCAGTTTAGGGGCA |  |
| 03846-qRT-F | TCCAGGTACGGGTGTTTGTG | Quantification of the VVMO6_03846 expression |
| 03846-qRT-R | AAGCGCTGCTTTTTCAGTGG |  |
| 01967-qRT-F | TAGGCTTTGCTGACTGGACG | Quantification of the VVMO6_01967 expression |
| 01967-qRT-R | ACAGTCCACACAGACGACTT |  |
| 00672-qRT-F | AAATTCCCGCTTGGCTGAGA | Quantification of the VVMO6_00672 expression |
| 00672-qRT-R | CAAAGGTAAGTTTGCCGCCC |  |
| 01809-qRT-F | GCCGCCGCGTTATTTGTATT | Quantification of the VVMO6_01809 expression |
| 01809-qRT-R | ACGGTGGTGATTTGTTTGCG |  |
| 01964-qRT-F | GCTACCGCTGACACTCAACT | Quantification of the VVMO6_01964 expression |
| 01964-qRT-R | CAGCATCAAAACGCCAACCA |  |
| 00249-qRT-F | GTTGGAGAGGTTGTGCGAGA | Quantification of the VVMO6_00249 expression |
| 00249-qRT-R | CCAATTCCGCCAAAAAGGCA |  |
| 03947-qRT-F | TAGCGGCGACAATGAAACCT | Quantification of the VVMO6_03947 expression |
| 03947-qRT-R | CCCATCACCGCAAGGGTATT |  |
| 03881-qRT-F | ACAGCTGGTTCCAGAGTTGG | Quantification of the VVMO6_03881 expression |
| 03881-qRT-R | AACGGGTTTCACCCAAAGGT |  |
| For mutant construction | | |
| HMPA01-F | CTTGTCATCACTCTTCTCTCTTTGAATG | Deletion of *VvhmpA* ORF |
| HMPA01-R | TATGGATCCTTCAATTCGTTAGTTTGGTA |  |
| HMPA02-F | GAAGGATCCATATTGATCGGCAGTGAT | Deletion of *VvhmpA* ORF |
| HMPA02-R | GGCTCCTCTATGGTGAAGAAAGTCTAA |  |
| For mutant complementation | | |
| HMPA03-F | GGTACCGGTGGGCTCCTCTATGGTGA | Amplification of *VvhmpA* ORF |
| HMPA03-R | GAGCTCCTAAACCACTTTGTGTGGGC |  |
| For protein overexpression | | |
| HMPA04-F | CCATGGGCATGCTCAGCGAAAACACCATTAAC | Amplification of *VvhmpA* ORF for the purification of His_6_-tagged *Vv*HmpA |
| HMPA04-R | GTCGACCTAAACCACTTTGTGTGGGCCAAA |  |
| HMPA05-F | GGCAACATATGCTCAGCGAAAACACCATTAC | Amplification of *VvhmpA* ORF for the purification of non-His_6_-tagged *Vv*HmpA |
| HMPA05-R | GGCCTCGAGCTAAACCACTTTGTGTGGGC |  |

^†^ The oligonucleotides were designed using the *V. vulnificus* MO6-24/O genome sequence (GenBank^TM^ accession numbers: CP002469 and CP002470).

^‡^ Regions of oligonucleotides not complementary to the corresponding genes are underlined.
