# Supplementary figures and images for: Transcriptomic Identification and Biochemical Characterization of HmpA, a Nitric Oxide Dioxygenase, Essential for Pathogenesis of Vibrio vulnificus
Source: Front Microbiol. 2019 Sep 24;10:2208. doi: 10.3389/fmicb.2019.02208 (PMC6768983; doi:10.3389/fmicb.2019.02208)

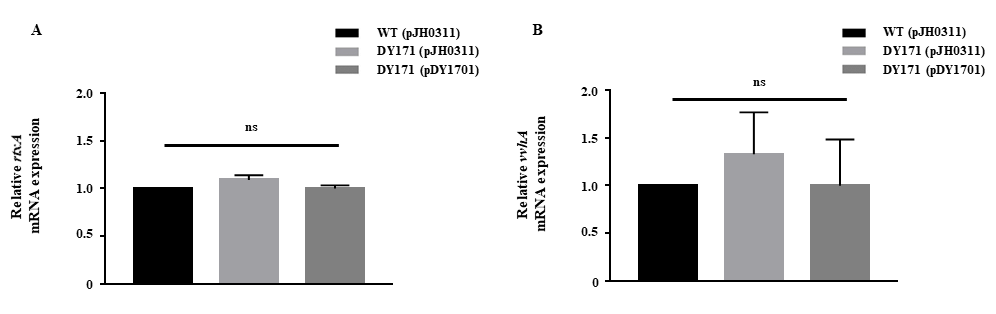

Supplement: Supplementary file 4 [file Image_1.TIF]
